# A Call to Address AI “Hallucinations” and How Healthcare Professionals Can Mitigate Their Risks

**DOI:** 10.7759/cureus.44720

**Published:** 2023-09-05

**Authors:** Rami Hatem, Brianna Simmons, Joseph E Thornton

**Affiliations:** 1 Department of Psychiatry, University of Florida College of Medicine, Gainesville, USA

**Keywords:** ethics, misinformation, artificial intelligence, chatgpt, hallucinations, mental health, psychiatry, ai & robotics in healthcare

## Abstract

Artificial intelligence (AI) has transformed society in many ways. AI in medicine has the potential to improve medical care and reduce healthcare professional burnout but we must be cautious of a phenomenon termed "AI hallucinations"and how this term can lead to the stigmatization of AI systems and persons who experience hallucinations. We believe the term "AI misinformation" to be more appropriate and avoids contributing to stigmatization. Healthcare professionals can play an important role in AI’s integration into medicine, especially regarding mental health services, so it is important that we continue to critically evaluate AI systems as they emerge.

## Editorial

Generative artificial intelligence (AI) has captivated the world by storm and revolutionized society in incomprehensible ways. AI chat boxes are designed to be easy to use, enhance our access to information, and improve our productivity as a society. While integrating AI services like ChatGPT (OpenAI, San Francisco, CA), Claude 2 (Anthropic, San Francisco), or Bard (Google, Mountain View, CA) into medicine can support mental health care and clinical decision-making, its ability to analyze large datasets and improve diagnostic accuracy and efficiency is not free of cost. Without careful consideration and monitoring by human healthcare professionals, AI algorithms, researchers, and practitioners can perpetuate existing biases, leading to unequal access to care, misdiagnoses, and inadequate treatment recommendations [1]. We call for each user of AI in healthcare to do their part in mitigating AI-generated misinformation. 

What are AI hallucinations?

AI hallucinations, as defined by ChatGPT3.5 (August 16, 2023),

 "[...] refer to the generation of content that is not based on real or existing data but is instead produced by a machine learning model's extrapolation or creative interpretation of its training data. These hallucinations can manifest in various forms, such as images, text, sounds, or even video. AI hallucinations occur when a machine learning model, particularly deep learning models like generative models, tries to generate content that goes beyond what is has learned from its training data. These models learn patterns and correlations from the data they are trained on and attempt to produce new content based on those patterns. However, in some cases, they can generate content that seems plausible but is actually a blend of various learned elements, resulting in content that might not make sense or could even be surreal, dream-like, or fantastical."

This can have consequences in healthcare as we begin to embrace AI as a tool. If a healthcare professional is unaware of AI’s limitations (i.e. AI hallucinations), they may inadvertently cause harm to patients due to inaccurate claims. 

In an editorial written by Dr. Hussam Alkaissi and Dr. Samy McFarlane to Cureus, they highlight several instances of AI hallucinations and how this can have implications in healthcare [2]. For example, when tasked to provide information on homocystinuria-associated osteoporosis and, on a separate occasion, late-onset Pompe disease, the AI provided a thorough paper with several citations with PubMed IDs. However, after fact-checking, it was found that the provided paper titles were fabricated and the PubMed IDs were associated with other papers [2]. Moreover, in an interview on CBS News’ “60 Minutes”, Google’s developers give their take on the future of AI and AI hallucinations. When Google’s Bard was tasked with writing an essay and recommending five books, it produced five seemingly realistic titles. However, the book titles were nothing but figments of an imaginative realm. They referred to these “hallucinations” as “errors with confidence” [3].

Alternative to the terminology of AI hallucinations

Initially, we proposed the term "AI confabulations" in a letter responding to Dr. Teva Brender’s Hey Chatbot, Write Me an H&P. In his reply, he agreed with the sentiment of abandoning the term "AI hallucinations", however, he thoughtfully noted that the word "confabulations" would arguably animate or attribute lifelike properties, further raising concerns about sentience [4-5]. We appreciate Dr. Brender for his comments and commitment to not perpetuating harmful stereotypes. We offer the alternative term "AI misinformation" as we believe it to be more accurate and in line with a shared vocabulary that individuals without AI knowledge can understand. Moreover, this term refrains from stigmatizing a community and avoids attributing lifelike characteristics to AI.

ChatGPT’s Response

The following is an excerpt from a ChatGPT3.5 conversation.

"Q: What would be an accurate word to describe when a large language model (LLM) generates false information?

A: An accurate word to describe when a large language model (LLM) generates false information is "misinformation." Misinformation refers to the dissemination of false or incorrect information, whether intentionally or unintentionally. If the false information is spread with the intention to deceive, the term "disinformation" could also be used."

How can healthcare professionals mitigate AI misinformation? 

When healthcare professionals incorporate AI into their services, they must exercise caution and recognize that AI alone cannot replace the individualized care that professionals offer. Even ChatGPT stresses the importance of “the human element of healthcare” and recognizes AI as a valuable tool in healthcare, not a replacement.

"AI can complement healthcare professionals by handling routine tasks, assisting in diagnosis, and improving treatment plans. However, the human element of healthcare-empathy, judgment, ethical decision-making, and adaptability-remains irreplaceable. The optimal approach is likely to involve a synergy between AI and human healthcare providers, with AI supporting and enhancing the work of professionals while leaving the core aspects of patient care in the hands of trained experts." - ChatGPT 3.5, August 16, 2023

To minimize the AI misinformation phenomenon, healthcare professionals should be aware of AI’s limitations and latest advancements. Some universities and hospitals provide free online continuing medical education (CME) courses, which now expand to include the scope of AI in healthcare. In clinical settings, AI-generated responses should be verified with reliable peer-reviewed medical sources. If any instances of AI misinformation are noted, they should be reported to the appropriate entity to ensure that these inaccuracies can be corrected. 

Large language models (LLM) such as ChatGPT 3.5 are only as accurate as the information provided to them; therefore, clinicians and AI scientists need to work together to continuously improve the data available to these systems. AI misinformation can be further mitigated by advocating for more diverse and representative datasets that would provide more generalizable data for these LLMs. While embracing the potential impact of AI in healthcare, it is important that we reiterate the importance of collaborating with patients and other colleagues to continue to bolster shared decision-making. 

Conclusion

We continue to believe the term "AI hallucination" is inaccurate and stigmatizing to both AI systems and individuals who experience hallucinations. Because of this, we suggest the alternative term "AI misinformation" as we feel this is an appropriate term to describe the phenomenon at hand without attributing lifelike characteristics to AI. As healthcare professionals begin to explore AI for clinical use, it is important we use it responsibly to ensure we do no harm. 
